# Central Nervous System Remyelination: Roles of Glia and Innate Immune Cells

**DOI:** 10.3389/fnmol.2019.00225

**Published:** 2019-09-19

**Authors:** Charbel S. Baaklini, Khalil S. Rawji, Greg J. Duncan, Madelene F. S. Ho, Jason R. Plemel

**Affiliations:** ^1^Department of Medicine, Division of Neurology, Neuroscience and Mental Health Institute, Faculty of Medicine & Dentistry, University of Alberta, Edmonton, AB, Canada; ^2^Wellcome Trust-Medical Research Council, Cambridge Stem Cell Institute, Cambridge Biomedical Campus, University of Cambridge, Cambridge, United Kingdom; ^3^Department of Neurology, Jungers Center for Neurosciences Research, Oregon Health and Science University, Portland, OR, United States

**Keywords:** remyelination, microglia, oligodendrocyte, oligodendrocyte progenitor cells, astrocytes, white matter disease, aging

## Abstract

In diseases such as multiple sclerosis (MS), inflammation can injure the myelin sheath that surrounds axons, a process known as demyelination. The spontaneous regeneration of myelin, called remyelination, is associated with restoration of function and prevention of axonal degeneration. Boosting remyelination with therapeutic intervention is a promising new approach that is currently being tested in several clinical trials. The endogenous regulation of remyelination is highly dependent on the immune response. In this review article, we highlight the cell biology of remyelination and its regulation by innate immune cells. For the purpose of this review, we discuss the roles of microglia, and also astrocytes and oligodendrocyte progenitor cells (OPCs) as they are being increasingly recognized to have immune cell functions.

## Introduction

Demyelination is a common feature of disease occurring after conditions such as spinal cord injury (SCI; Crowe et al., 1997; Powers et al., 2012; Plemel et al., 2014), multiple sclerosis (MS; Franklin and Ffrench-Constant, 2008; Plemel et al., 2017), stroke (Rosenzweig and Carmichael, 2015; Khodanovich et al., 2018), and traumatic brain injury (Mierzwa et al., 2015; Armstrong et al., 2016). Moreover, white matter loss occurs in Alzheimer’s disease (Mitew et al., 2010; Carmeli et al., 2013; Zhan et al., 2014; Bejanin et al., 2017) and is a feature of aging that is correlated with cognitive decline (Bartzokis et al., 2012; Yeatman et al., 2014; Li et al., 2017). With age, myelin shows increasing signs of damage while at the same time myelin debris accumulates within microglia (Safaiyan et al., 2016; Hill et al., 2018), which can trigger cholesterol crystal formation and inflammasome activation (Cantuti-Castelvetri et al., 2018). In MS, more remyelination is associated with less disability (Bramow et al., 2010; Bodini et al., 2016), suggesting that enhancing remyelination is a viable therapeutic strategy that is only recently showing benefits in clinical trials (Plemel et al., 2017). However, for those with MS, remyelination is variable and prone to failure (Prineas et al., 1993; Patrikios et al., 2006; Patani et al., 2007; Bramow et al., 2010), especially in the context of aging which is known to slow the rate of remyelination (Shields et al., 1999; Goldschmidt et al., 2009; Ruckh et al., 2012; Brown et al., 2014; Frischer et al., 2015). In this review article, we focus on the innate immune cells of the central nervous system (CNS) and their role during remyelination. We include microglia as the primary CNS innate immune cells, but also recognize that other glia such as astrocytes and oligodendrocyte progenitor cells (OPCs) may sculpt the innate immune response.

## Cell Biology of Remyelination

### Importance of Remyelination

Demyelination, or the loss of myelin, can be induced due to injury or disease. MS is characterized by inflammatory demyelination, possibly induced by monocytes, macrophage/microglia, and T-cells. For example, microglia/macrophage-derived oxidative species cause damage by inducing myelin and axonal injury (Haider et al., 2011; Nikic et al., 2011; Witte et al., 2019). The immune system produces demyelination by many other mechanisms, but this is not the focus of this review (Mayo et al., 2012; Lassmann and van Horssen, 2016; Mishra and Yong, 2016; Davies and Miron, 2018). Demyelination slows axonal conduction, and as suggested by computer simulations (Koles and Rasminsky, 1972; Waxman and Brill, 1978), may even result in a failure to propagate the signal past the demyelinated segment (Koles and Rasminsky, 1972; Waxman and Brill, 1978). The conduction block associated with demyelination is thought to relate to ionic disbalance once the myelin is removed. The loss of myelin unmasks potassium channels underneath the myelin sheath that can act as a current sink (Schauf and Davis, 1974; Bostock et al., 1978, 1981; Sherratt et al., 1980; Wang et al., 1993, 1995). To compensate, axons can upregulate voltage-gated sodium channels along their length (Foster et al., 1980; England et al., 1991; Black et al., 2007; Hamada and Kole, 2015). Potentially due to this upregulation or redistribution of voltage-gated sodium channels, it is possible to propagate the signal along a demyelinated segment, albeit more slowly (Felts et al., 1997). Associated with the axonal conduction block is a corresponding functional loss or alteration. For example, demyelination of the ventral medial geniculate nucleus (vMGN) or A1 region in the auditory cortex in mice results in both impaired sound frequency-specific responses and an increase in latency in auditory responses (Narayanan et al., 2018). Importantly, while remyelination may speed axonal propagation, it is not always linked to functional recovery (Duncan et al., 2009, 2018; Mozafari et al., 2010; Assinck et al., 2017a; Narayanan et al., 2018). Both the extent and severity of demyelination may dictate the functional consequences. For example, in an experimental murine model of SCI where demyelination is confined to the lesion, preventing remyelination did not change the spontaneous recovery, potentially because after SCI conduction is not sufficiently disrupted (Felts et al., 1997; Duncan et al., 2017, 2018).

Remyelination also maintains axonal integrity and attenuates axonal degeneration. If remyelination is slowed using irradiation to ablate OPCs in mice, there is more axonal degeneration (Irvine and Blakemore, 2008). However, this study is confounded by off-target effects of irradiation including blood-brain barrier (BBB) disruption (Diserbo et al., 2002), enhanced astrogliosis (Wilson et al., 2009), and activation of microglia (Hwang et al., 2006; Irvine and Blakemore, 2008). More specifically, if remyelination is accelerated by promoting oligodendrocyte differentiation in a cell-type specific manner, more axons are preserved (Mei et al., 2016). How might myelin protect axons? One likely mechanism is *via* the buffering of potassium through the potassium channel KIR4.1, expressed in oligodendrocytes (Schirmer et al., 2018). Potassium is released from axons as part of their action potential and conditional oligodendrocyte-specific removal of this KIR4.1 buffering results in late-onset axonal degeneration, suggesting that myelinic potassium buffering maintains axonal integrity. Oligodendrocytes also support axons by providing them with glycolytic metabolites *via* the myelin sheath (Fünfschilling et al., 2012; Saab et al., 2016; Micu et al., 2017). Even the myelin sheath may itself can act as a protective barrier by surrounding the axon from toxic reactive oxygen species (Nikic et al., 2011; Witte et al., 2019). The myelin sheath also shifts some of the metabolic demands from the axon to the oligodendrocyte. For example, when an axon is myelinated there is less sodium released for an axon potential, and therefore less energy is required to repolarize its membrane, yet the production of myelin is energetically expensive (Harris and Attwell, 2012). Other possible mechanisms of axonal support by oligodendrocytes include the release of oligodendrocyte-derived exosomes or ribosomes (Frühbeis et al., 2013; Shakhbazau et al., 2016). Oligodendrocytes can also secrete many other factors to boost neuronal health or survival in culture such as insulin-like growth factor 1 (IGF-1) and glial cell-derived neurotrophic factor (GDNF; Wilkins et al., 2001, 2003; Dai et al., 2003), which may support axons *in vivo*.

An analogous relationship between glial cells and axons exists in the peripheral nervous system (PNS). Schwann cells (SCs) are glial cells generally restricted to the PNS that arise from neural crest-derived SC precursors (Monk et al., 2015). Under normal conditions, SCs myelinate axons in the PNS. However, there is evidence that SCs can remyelinate in the CNS under inflammatory conditions. In fact, P_0_ staining—a marker for SC-derived myelin sheaths—was observed in MS spinal cord lesions (Itoyama et al., 1983). Indeed, fate-mapping revealed that in LPC-demyelinated lesions, PDGFRa and NG2-expressing progenitor cells in the CNS produce the myelinating oligodendrocytes and SCs (Zawadzka et al., 2010).

### Process of Remyelination

Remyelination is a regenerative process that requires the sequential activation and recruitment of OPCs to areas of demyelination, followed by their differentiation into new oligodendrocytes—but also less frequently into SCs—and subsequent myelin deposition around demyelinated axons (Franklin and Ffrench-Constant, 2008, 2017; Zawadzka et al., 2010; Plemel et al., 2017). OPCs are found throughout the adult mouse brain white matter and gray matter (Dimou et al., 2008; Rivers et al., 2008; Kang et al., 2010). After a demyelinating insult, OPCs transition to a reactive state with the upregulation of key transcription factors such as Nkx2.2, Olig2 and Sox2 (Levine and Reynolds, 1999; Fancy et al., 2004; Zhao et al., 2015). In the lesion, there is a release of many growth factors such as platelet-derived growth factor (PDGF) and fibroblast growth factor (FGF; Hinks and Franklin, 1999; Messersmith et al., 2000). These factors and others are thought to regulate OPC lineage progression by increasing proliferation and migration towards the lesion (Messersmith et al., 2000; Murtie et al., 2005; Dehghan et al., 2012). OPCs proliferate and migrate in the days following demyelination or injury, as part of their recruitment (Redwine and Armstrong, 1998; Hughes et al., 2013). Following OPC recruitment, they differentiate and mature into myelinating oligodendrocytes, which involves the ensheathment and enwrapping of the denuded axon with a new myelin sheath (Tripathi et al., 2010; Zawadzka et al., 2010; Crawford et al., 2016b). Differentiated oligodendrocytes start expressing myelin proteins such as myelin basic protein (MBP), which allows for the compaction of myelin membranes (Kimura et al., 1989; Watanabe et al., 2002; Polito and Reynolds, 2005; Snaidero et al., 2014). Much of the process of oligodendrocyte differentiation is due to key transcription factors. For example, the transcription factor myelin regulatory factor (MyRF) is required for oligodendrocyte maturation and myelination (Emery et al., 2009) and effective remyelination in adulthood (Duncan et al., 2017). MyRF binds to enhancer sequences and directly drives the expression of myelin genes (Bujalka et al., 2013); it is necessary for remyelination after chemical and traumatic demyelinating injuries (Duncan et al., 2017, 2018). Remyelinating oligodendrocytes can adopt different phenotypes, as shown by single-nucleus RNA sequencing (Jäkel et al., 2019). Interestingly, oligodendrocytes in the white matter of MS patients compared to non-diseased controls have different subclustering based on their transcriptomes. This shift in oligodendrocyte phenotypes may reflect an impaired capacity to remyelinate but the functional consequences of these different phenotypes have not yet been directly determined.

Much of the research on remyelination is conducted on demyelinating rodent models. Using these models, it has been shown that OPCs, and not mature oligodendrocytes, are the cells most responsible for remyelination (Crawford et al., 2016a). Transplantation of mature oligodendrocytes into demyelinated lesions do not remyelinate denuded axons (Targett et al., 1996). Oligodendrocyte fate-mapping in mice also demonstrated that mature oligodendrocytes do not engage in remyelination (Crawford et al., 2016a). Surprisingly, new data provides evidence that mature oligodendrocytes can remyelinate in larger mammals. Duncan et al. (2018) find the presence of oligodendrocytes with both thick and thin myelin sheaths using a feline irradiated food-induced demyelination (FIDID) model and vitamin B12 deficiency and in a non-human primate model. Given that remyelination is typically, but not always associated with thin myelin sheaths (Blakemore, 1974; Duncan et al., 2017), one explanation for these results is that spared oligodendrocytes with a thick, unaffected, myelin sheath can re-extend their processes and remyelinate adjacent denuded axons (Duncan et al., 2018). An alternative possibility with this study is that myelin sheath thickness may be differentially regulated by the axon. In a recent study, Yeung et al. (2019) investigated oligodendrogenesis in humans with MS using a specific carbon dating strategy. After cell division, the daughter cell’s DNA will have a radioactive carbon concentration that is proportional to concentration of radioactive carbon in the atmosphere. As a result of atomic bomb testing starting in the mid-late 1940s, the atmospheric radioactive carbon increased making it possible to date daughter cells after the 1940s based on the nuclear radioactive carbon levels. Using this strategy, Yeung et al. (2019) measured new oligodendrocyte production in shadow plaques, which are regions of intermediate lipid staining that are thought to be remyelinated. Surprisingly, they found that new oligodendrocyte production was similar in shadow plaques to those found in the non-diseased brain, suggesting that in humans the mature oligodendrocytes can remyelinate denuded axons. However, in this one study, these conclusions are based off of 11 samples. Moreover, this study found that OPCs did turn over in shadow plaques, but due to the levels of atmospheric radioactive carbon, this was only apparent for those born in the mid-1960s onwards. Only one shadow plaque was measured for oligodendrocyte carbon after this critical mid-1960s period. The question of whether adult oligodendrocytes can remyelinate will, therefore, require further validation in future studies.

## Roles of Microglia and Infiltrating Macrophages in Remyelination

Microglia are immunocompetent glial cells of the CNS (Streit, 2002). They are the CNS parenchymal macrophages that arise developmentally from erythromyeloid precursors in the yolk sac (Ginhoux et al., 2010; Kierdorf et al., 2013). They are unevenly distributed according to the CNS region and range from 5 to 12% of glia in the mouse brain and 0.5%–16.6% of human brain parenchymal cells (Lawson et al., 1990; Mittelbronn et al., 2001). They are important for controlling many neurodevelopmental processes. For example, neural precursor cells proliferate in the presence of microglia *in vitro* (Antony et al., 2011). Also, microglia conditioned media promotes the differentiation of neural precursor cells into neurons as well as astrocytes (Nakanishi et al., 2007; Antony et al., 2011). Microglial ablation results in neuronal apoptosis and a decrease in spine density in young mice indicating microglia promote synaptogenesis and the survival of neurons (Ueno et al., 2013; Miyamoto et al., 2016). Microglia also regulate myelinogenesis through the secretion of growth factors like IGF-1, which is critical for *Mbp* expression in young mice (Wlodarczyk et al., 2017).

### Microglia Response to Injury

Microglia regulate homeostasis by surveying their microenvironment but are highly responsive to injury or disease as laser-induced injury in the mouse neocortex results in microglial extensions surrounding the site of injury (Davalos et al., 2005; Nimmerjahn et al., 2005). When there is more damage over a longer period of time, for example following focal demyelination with LPC, microglia can retract their processes and become more spheroidal (Plemel et al., 2018). These morphological attributes of activated microglia, as well as similar expression patterns, have made it difficult to differentiate microglia from other macrophages such as border-associated macrophages in the CNS that include meningeal, choroid plexus and perivascular macrophages (Goldmann et al., 2016; Mrdjen et al., 2018), as well as monocyte-derived macrophages (Butovsky et al., 2014). Most studies do not differentiate between these cell types. As such, in this review article, these cells will be referred to as microglia/macrophages.

Microglia are surveillant cells that are highly responsive to environmental cues. In adults, microglia self-renew with modest proliferation (Nimmerjahn et al., 2005; Elmore et al., 2014; Kawabori and Yenari, 2015). In the uninjured state of the CNS, *in vivo* imaging revealed that ramified microglia continuously scan their microenvironment by undergoing structural changes including filopodia extension and retraction (Nimmerjahn et al., 2005; Bernier et al., 2019). By this surveillance mechanism, using two-photon microscopy of living murine microglia, Davalos et al. (2005) demonstrate that they detect and act accordingly to damage-associated molecular patterns (DAMPs). Microglia respond to disease conditions through a combination of receptors such as pattern recognition receptors, purinergic and fractalkine receptors, and cytokine receptors (Hickman et al., 2013). Microglia likely responds to hundreds, if not thousands of molecules, many in undefined ways. Certain molecules elicit specific responses, for example, the subsequent activation of purinergic receptors leads to the activation of the phagocytic pathway in rat microglia, that involves the clearance of apoptotic cells, both *in vitro* and *in vivo* (Davalos et al., 2005; Haynes et al., 2006; Koizumi et al., 2007; Bernier et al., 2019).

### Microglia During Remyelination

In the context of remyelination, microglia/macrophages can have many beneficial roles. However, these cells also contribute to autoimmune-related toxicity (Heppner et al., 2005; Ajami et al., 2011; Goldmann et al., 2013; Rothhammer et al., 2018). The dichotomous nature of microglia and macrophages is incompletely understood. One important function of microglia/macrophages following demyelination is the removal of inhibitory myelin debris present in the lesion. This myelin debris has been shown to negatively regulate OPC lineage progression (Syed et al., 2008; Plemel et al., 2013) and consequently, remyelination (Kotter et al., 2006). As microglia are phagocytotic cells, they contribute to myelin debris clearance (Sosa et al., 2013; Rawji et al., 2018). This phagocytosis is reflected by the presence of myelin proteins such as MBP in microglia/macrophages in the white matter following demyelination (Sosa et al., 2013).

Microglia also secrete an array of signaling molecules, including cytokines, chemokines and growth factors; many of these factors signal *via* receptors on oligodendrocyte lineage cells. For instance, the proinflammatory cytokine tumor necrosis factor-α (TNF-α), secreted by microglia/macrophages as well as astrocytes in mouse cuprizone-induced lesions, promotes OPC expansion and remyelination through TNF receptor 2 (TNFR2) on NG2^+^ cells (Arnett et al., 2001; Voss et al., 2012). Interleukin-1β (IL-1β), also secreted by microglia/macrophages and astrocytes, is important for remyelination potentially by controlling oligodendrocyte lineage cell’s survival by stimulating IGF-1 secretion from microglia/macrophages in cuprizone-treated mice (Mason et al., 2001). Recently, it was shown microglia can modulate remyelination through the secreted enzyme transglutaminase 2 (TG2). TG2 interacts with OPC-specific Adhesion G Protein-Coupled Receptor G1 (ADGRG1) to promote OPC proliferation. Either, microglia-specific loss of TG2 or OPC-specific loss of ADGRG1 impairs remyelination in two murine demyelination models (Giera et al., 2018). As microglia/macrophages transition from a more pro-inflammatory state in the days after demyelination to a more immunoregulatory phenotype at a later time point, they secrete Activin-A in mouse lesions (Miron et al., 2013). Activin-A release, in turn, interacts with activin receptors on OPCs, which are required for developmental myelination and remyelination (Dillenburg et al., 2018).

It is important to note that some of the cytokines that have pro-remyelinating roles can be implicated in mediating pathogenesis, including, for example, TNF-α that is required for autoimmune demyelination (Ruddle et al., 1990; Selmaj et al., 1991; Baker et al., 1994; Steeland et al., 2017). In addition, IL-1β from neutrophils and monocyte-derived macrophages drives neuroinflammation in EAE mice (Lévesque et al., 2016; Paré et al., 2018). Indeed, in autoimmune demyelination the removal of a key NF-kB regulator, TAK1, from microglia and CNS macrophages prevents EAE, suggesting an important role of these cells in the execution of autoimmune demyelination (Goldmann et al., 2013).

### Microglia Response Is Heterogeneous

Recent transcriptomic analyses in both mice and humans revealed that the microglia response to acute demyelination is more heterogeneous than previously thought (Hammond et al., 2019; Masuda et al., 2019). In the resting state, microglia typically adopt a homeostatic phenotype characterized by the expression of such markers as CX3CR1, the purinergic receptor P2RY12, and TMEM119 (Krasemann et al., 2017; Masuda et al., 2019). As microglia become activated, they shift their phenotype. One recently characterized microglia phenotype is the damage-associated microglia (DAM), which are a subset of microglia revealed by single-cell RNA sequencing in Alzheimer’s Disease transgenic mice (Keren-Shaul et al., 2017). DAM are linked to neurodegeneration and are characterized by the upregulation TREM2, a phagocytic marker (Deczkowska et al., 2018). Many of these markers associated with DAM are also upregulated in microglia following demyelination such as *Lpl, Cst7* and *Apoe*, suggesting that there is an overlapping response between neurodegeneration and demyelination (Keren-Shaul et al., 2017; Hammond et al., 2019). The transition to a less pro-inflammatory microglia/macrophage phenotype is promoted by the secreted enzyme, interleukin 4 induced 1, which is induced in microglia in response to interleukin 4 (Psachoulia et al., 2016). Recently, it was shown that necroptosis of microglia/macrophages is important in the phenotypic shift during remyelination, suggesting that microglia/macrophages may not transition between phenotypes but instead die, only to be replaced by alternative phenotypes (Lloyd et al., 2019).

### Infiltrating Macrophages During Remyelination

The functional overlap of monocytes-derived macrophages and microglia during remyelination is still unclear. Monocyte-derived macrophages are mononuclear phagocytic cells of the hematopoeitic stem cell lineage (Jakubzick et al., 2017). Under inflammatory conditions in the CNS, circulating monocytes cross the BBB and mature into macrophages (King et al., 2009; Caravagna et al., 2018). These infiltrating macrophages are evident in both MS lesions and in the different models of demyelination/remyelination such as EAE, LPC and cuprizone-treated animal models (Hiremath et al., 1998; Trebst et al., 2001; King et al., 2009; Miron et al., 2013; Vogel et al., 2013; Kuhlmann et al., 2017). Monocytes that express C-C Motif Chemokine Receptor (CCR2), known as inflammatory monocytes, migrate into the CNS in response to C-C Motif Chemokine Ligand 2 (CCL2). Following cuprizone toxicity or in aged mice following focal demyelination, the vast majority of monocytes require CCR2 for their entry (Ruckh et al., 2012; Lampron et al., 2015). CCR2-knockout mice are resistant to EAE (Fife et al., 2000) and blocking monocyte infiltration prevents EAE in mice (Ajami et al., 2011) suggesting that monocytes are required for autoimmune-mediated demyelination in part by the production of reactive oxygen species (Nikic et al., 2011; Locatelli et al., 2018).

The roles of monocyte-derived macrophages are just beginning to be characterized during remyelination and can be difficult to delineate from microglia as experimentally manipulating these cells while leaving the microglial response intact is challenging. During remyelination, the early use of clodronate liposomes reduces remyelination. Clodronate liposomes are artificial lipid vesicles that are taken up preferentially by phagocytic cells and kill circulating monocytes and macrophages (van Rooijen, 1992; Van Rooijen and Sanders, 1994; Popovich et al., 1999; Kotter et al., 2006; Döring et al., 2015), but they can also ablate microglia (Kumamaru et al., 2012; Han et al., 2019). It is, therefore, still unclear whether clodronate liposomes impair remyelination by killing microglia or macrophages or both. One important correlate of remyelination is the removal of inhibitory myelin debris for which young monocyte-derived macrophages participate (Ruckh et al., 2012). However, it is still unclear whether microglia or macrophages predominate during myelin debris phagocytosis. Taken together, CNS-infiltrating macrophages promote demyelination, but the overlap in functions between microglia and macrophages during remyelination is less clear.

**Figure 1:**
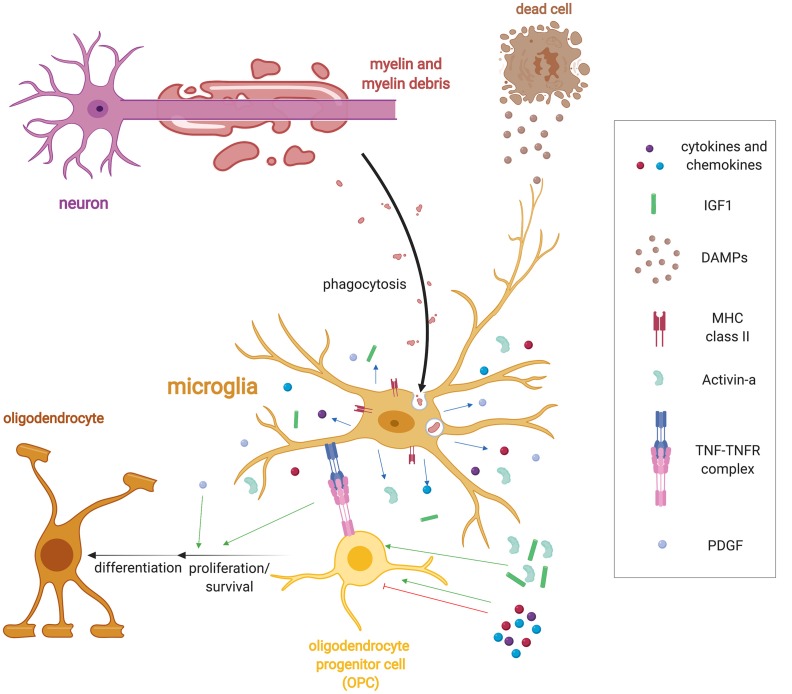
Microglia regulation of remyelination. Following demyelination, microglia detect injury in the form of damage associated molecular patterns (DAMPs) *via* a variety of receptor classes such as pattern recognition receptors, purinergic and fractalkine receptors, and cytokine receptors. DAMPS induce a wide range of functions including the activation of microglia to phagocytose myelin and cellular debris. Microglia also upregulate a host of immune molecules when activated such as MHC-II expression and release cytokines, chemokines and growth factors such as Activin-A, platelet-derived growth factor (PDGF), IL-1β, and IGF-1 that regulate oligodendrocyte progenitor cell (OPC) lineage progression. They also express tumor necrosis factor-α (TNF-α), which binds to TNF receptor 2 (TNFR2) to promote remyelination.

Taken together, microglia and macrophage likely possess overlapping functions that are not completely defined, which as a whole are required for remyelination (Figure 1). One critical function is the phagocytosis of inhibitory myelin debris, but other key roles of these cells include secretion of growth factors and the regulation of the immune response through cytokines and other immunoregulatory molecules. The response of these cells is heterogeneous and exciting new technologies such as single-cell RNA sequencing will allow for the characterization of their response during the continuum of remyelination.

## Roles of Other Innate Immune Cells During Remyelination

Following demyelination microglia/macrophages predominate, but other innate immune cells could potentially participate in remyelination. The other cells of the innate immune system include natural killer cells, mast cells, eosinophils, basophils, neutrophils, and dendritic cells. Many of these innate immune cells can inflict tissue damage (Mayo et al., 2012; Boutajangout and Wisniewski, 2013; Courties et al., 2014), but little is known about their role in remyelination. In the context of demyelinating diseases, several of these innate immune cells have a described role during demyelination. For instance, neutrophils are important players in disease pathogenesis as mice lacking the key neutrophils chemokine receptor, C-X-C motif chemokine receptor 2 (CXCR2), are resistant to cuprizone-induced oligodendrocyte cell death and demyelination (Liu et al., 2010). Eosinophils also contribute to demyelination as preventing their infiltration into the CNS in EAE mice decreases disease severity (Gladue et al., 1996). Mast cells, which are found in MS lesions, are also important in disease pathogenesis as blocking their ability to degranulate in EAE rats reduces disease severity (Olsson, 1974; Dimitriadou et al., 2000). To date, less is known about how eosinophils, basophils and neutrophils regulate remyelination. Dendritic cells, which are professional antigen producing cells, may have pro-remyelinating roles (Pusic et al., 2014). Dendritic cells challenged with interferon γ (IFNγ) release exosomes containing microRNA such as miR-219. These dendritic cell-derived exosomes improve remyelination in cultured rat hippocampal slices *ex vivo*, however, it is still unclear how dendritic cells regulate remyelination *in vivo*. Given the complex interplay between various innate immune cells and their known involvement in repair of other systems, such as during wound healing (MacLeod and Mansbridge, 2016), there are likely other unknown roles for these cells during remyelination.

## Roles of Astrocytes in Remyelination

Astrocytes have important roles in both CNS development and homeostasis such as during synaptogenesis, neurotransmission, BBB formation and maintenance, among other roles (Sofroniew and Vinters, 2010; Molofsky and Deneen, 2015). In response to neuroinflammation, astrocytes respond in a process called reactive astrogliosis (Zamanian et al., 2012). Reactive astrocytes have both potentially beneficial and detrimental roles during remyelination; indeed, these functions may relate to a particular phenotype that the astrocytes adopt (Liddelow and Barres, 2017; Liddelow et al., 2017). Astrocytes may promote/inhibit remyelination directly, but could also signal through microglia to promote remyelination. For example, the ablation of reactive astrocytes in cuprizone-treated mice impairs recruitment of microglia to the demyelinating lesion (Skripuletz et al., 2013). Lowered microglia recruitment is associated with reduced clearance of inhibitory myelin debris, which impairs oligodendrocyte maturation (Syed et al., 2008; Plemel et al., 2013) and remyelination (Kotter et al., 2006). The inverse is likely also true, whereby microglia can regulate astrocyte function. Microglia activated by a TLR4 agonist, LPS, induces a newly identified neurotoxic phenotype of astrocytes. The neurotoxic astrocyte may be detrimental for OPC maturation as it has been shown to induce oligodendrocyte and neuronal apoptosis (Liddelow et al., 2017). As microglia/macrophages display a temporal shift from a predominantly pro-inflammatory phenotype to an anti-inflammatory phenotype (Miron et al., 2013), it is unknown whether the astrocytes within a demyelinating lesion adopt a similar temporal shift in phenotype and whether this may in turn yield a spectrum of beneficial or detrimental functions for remyelination.

One role of astrocytes is mediated by endothelin-1, a peptide that has been characterized to potently induce vasoconstriction. Following demyelination in animal models and in MS patients’ lesions, astrocytes begin expressing endothelin-1 (ET-1; Hammond et al., 2014). ET-1 is secreted by astrocytes and endothelial cells and acts in paracrine and autocrine manners in the CNS (Hostenbach et al., 2016). ET-1 can activate receptors on vascular smooth muscle cells, inducing vasoconstriction and leading to cerebral hypoperfusion, which is commonly observed in MS patients (Law et al., 2004; Varga et al., 2009). Low oxygen tension, at least during development in mice, also impairs OPC maturation (Yuen et al., 2014). Following acute demyelination, astrocytes respond to ET-1 *via* their endothelin receptor B by upregulating the Notch1 receptor ligand, jagged1 (Hammond et al., 2014, 2015). Astrocyte-induced Notch1 activation is known to inhibit OPC differentiation and remyelination. Interestingly, this astrocyte jagged1-Notch1 interaction resolves to allow OPC differentiation after a period of OPC expansion. Astrocytes could, therefore, be delaying OPC differentiation to allow sufficient proliferation of OPCs (Zhang et al., 2009; Hammond et al., 2014). This notch-mediated interaction may be relevant to MS considering a histological analysis of lesions shows that astrocytes express jagged1 and OPCs express Notch1 (John et al., 2002). This is important as it is thought that a differentiation and maturation failure of OPCs is a significant contributor to remyelination failure in MS lesions (Kuhlmann et al., 2008; Duncan et al., 2017).

### Astrocytes as Regulators of the Extracellular Matrix (ECM)

Astrocytes are also major modulators of the extracellular matrix (ECM). The ECM is composed of several molecules which can influence OPC function (Pu et al., 2018). Astrocytes are major producers of the ECM molecules high molecular weight hyaluronan (Back et al., 2005), Tenascin-C (Gutowski et al., 1999), fibronectin (Stoffels et al., 2013), and various members of the chondroitin sulfate proteoglycan family (Jones et al., 2003; Siebert et al., 2014). In addition to providing chemical signals, the mechanical stiffness of the ECM significantly influences the proliferation and differentiation of OPCs *in vivo*. It was recently shown that aging rodents have a stiffer ECM, resulting in decreased OPC proliferation and differentiation (Segel et al., 2019). When the mechanosensitive ion channel PIEZO1 is inhibited, OPCs are able to restore their loss of function in proliferation and differentiation. In addition to inhibiting OPC maturation and axon regeneration, these molecules exert immunomodulatory effects on several members of the immune system (Stephenson et al., 2018). For example, when chondroitin sulfate proteoglycans are added to murine macrophages *in vitro*, these cells significantly upregulate their production of pro-inflammatory cytokines, matrix metalloproteinases, as well as displaying an increase in migration (Stephenson et al., 2018). In this study, these ECM molecules were observed around perivascular cuffs in the EAE model. Pharmacologically inhibiting the synthesis of these molecules in this model resulted in reduced immune cell infiltration and decreased clinical severity (Stephenson et al., 2019). Although not yet addressed, the production of such inhibitory molecules may be influenced by the phenotypic state adopted by astrocytes.

### Astrocyte Role in Lipid Metabolism

Astrocytes play a central role in lipid metabolism. As the BBB limits the entry of several lipoproteins from the peripheral circulation, the CNS has developed a tightly regulated system in which to regulate the production of lipid species such as arachidonic acid, docosahexanoic acid, and cholesterol (Moore, 2001; Dietschy, 2009). Astrocytes are the major producers of cholesterol and the cholesterol carrier, apolipoprotein E (APOE) under homeostatic conditions (Boyles et al., 1985). Cholesterol is a major constituent of cell membranes and myelin and is therefore critical in normal CNS functioning (Linton et al., 1991). Rodent neurons do not readily synthesize cholesterol and depend on astrocytes for their source of cholesterol (Nieweg et al., 2009). As astrocyte-derived cholesterol is important for normal neuronal functioning, it is likely that transport of cholesterol to oligodendrocytes from astrocytes or other glial cells such as microglia is important in myelin synthesis. Indeed, it was recently shown that demyelinated white matter lesions from aging mice have deficient reverse cholesterol transport that was associated with reduced remyelination (Cantuti-Castelvetri et al., 2018). Cantuti-Castelvetri et al. (2018) found that stimulating reverse cholesterol transport enhanced remyelination in the aging CNS, highlighting the role of lipid metabolism in myelin synthesis. Whether cholesterol exported by astrocytes and macrophages/microglia can be directly taken up by OPCs responding to a demyelinated lesion was not addressed in this study and is yet to be determined. This area is of direct importance as several small molecules capable of stimulating oligodendrogenesis act on the cholesterol biosynthesis pathways (Hubler et al., 2018). Importantly GFAP-expressing reactive astrocytes in EAE mice have decreased cholesterol synthesis, which may limit remyelination efficiency (Itoh et al., 2018), suggesting that autoimmunity may impair cholesterol synthesis.

Medications used to treat hypercholesterolemia (statins) seem to show some benefit in secondary progressive MS (Chataway et al., 2014). High-dose statin treatment resulted in a 43% reduction in brain atrophy compared to placebo. As statins have effects on the immune system, it is unclear whether the therapeutic efficacy of statins in secondary progressive MS are mediated through cholesterol modulation or other indirect mechanisms (Eshaghi et al., 2019). It is important to note that statins did not influence relapse risk, disease progression or disability scores in people with MS in combination with interferon therapy (Bhardwaj et al., 2012).

### Astrocytes Regulate CNS Schwann Cell Myelin and Secrete Growth Factors

Astrocytes are thought to regulate the degree to which remyelination is favored by OPC-derived oligodendrocytes or SCs (Zawadzka et al., 2010; Monteiro de Castro et al., 2015). In the absence of astrocytes, more SC remyelination is observed. The mechanism by which astrocytes regulate the fate choice of OPCs is unclear but may be due to interactions in BMP/Wnt signaling within the lesion microenvironment (Ulanska-Poutanen et al., 2018). Although SCs remyelination appears to re-establish conduction capacity within CNS axons (Smith et al., 1979; Blight and Young, 1989), it is not yet clear whether SCs differ from oligodendrocytes in the provision of metabolic support to axons. Similarly, it is unclear how different myelin sheath thicknesses between oligodendrocytes and SCs affect the timing of axonal conduction.

Astrocyte secretion of neurotrophins, a class of proteins that induce the growth and survival of neuronal cells, are involved in remyelination. For example, astrocytes can regulate myelin protein synthesis by the release of brain-derived neurotrophic factor (BDNF; Fulmer et al., 2014). In the cuprizone model of demyelination, the activation of OPC-expressed BDNF receptor TrkB promotes remyelination as reflected by an increase in OPC differentiation, the number of remyelinated axons and myelin sheath thickness (Fletcher et al., 2018). Furthermore, astrocytes secrete the mitogen platelet-derived growth factor-A (PDGF-A) to act on OPCs, which can promote proliferation (Wolswijk and Noble, 1992; Redwine and Armstrong, 1998; Frost et al., 2003). Lesions from MS also display PDGFRα expression on proliferating cells (Maeda et al., 2001). Astrocytes are also thought to produce leukemia inhibitory factor, ciliary neurotrophic factor, and insulin-like growth factor-1, all of which have been implicated in supporting OPC maturation (Moore et al., 2011).

**Figure 2:**
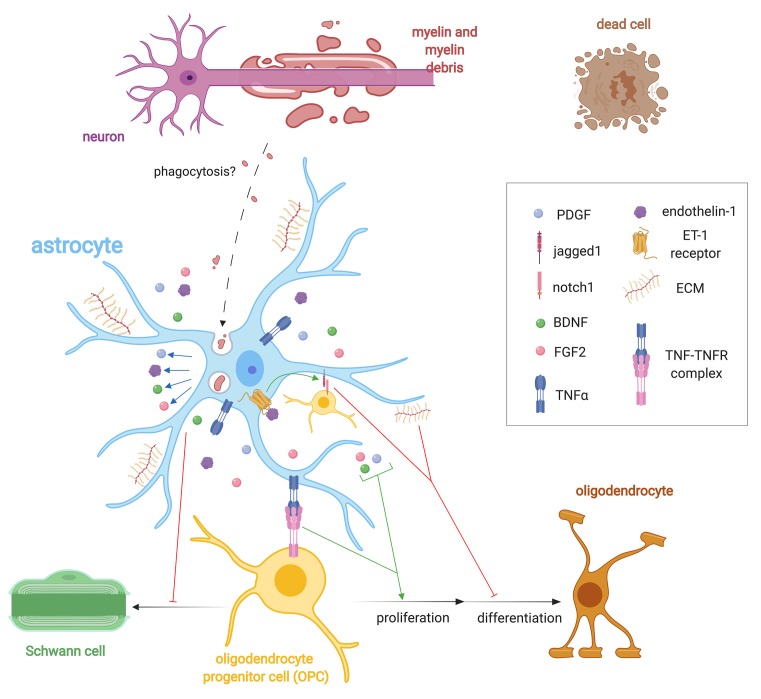
Importance of astrocyte regulation of remyelination. Following demyelination, astrocytes help regulate OPC behavior *via* the expression of extracellular matrix (ECM) molecules such as CSPGs, hyluronan, fibronectin, and tenacin C, endothelin-1 and growth factor secretion such as FGF2, brain-derived neurotrophic factor (BDNF), and PDGF. Endothelin-1, through autocrine and paracrine signaling, stimulates the expression of the notch ligand jagged1 that antagonizes remyelination. Astrocytes control the fate choice of OPCs following demyelination by antagonizing their differentiation into Schwann cells (SCs). Astrocytes may phagocytose myelin debris, but this has only been validated *in vitro*. They also express TNF-α, which binds to TNFR2 to promote remyelination.

Taken together, the role of astrocytes during remyelination is complex and incompletely understood (Figure 2). Release of growth factors and lipid metabolism is required for remyelination, yet astrocytes also produce several inhibitory ECM molecules that impair remyelination. The expression of notch ligands by astrocytes also serves as a break for remyelination. How astrocytes are regulated through the continuum of remyelination is yet to be defined, but may provide an explanation for their diverse roles during remyelination.

## Non-myelinating Roles of Oligodendrocyte Lineage Cells During Remyelination

The non-myelinating roles of oligodendrocyte lineage cells are being increasingly realized and may provide new avenues for therapeutic intervention. During development and into adulthood, OPC differentiation is restricted to forming mature oligodendrocytes (Kang et al., 2010; Young et al., 2013; Huang et al., 2019) but during traumatic injury or demyelination OPCs act as multipotent progenitor cells (Richardson et al., 2011; Crawford et al., 2014) capable of differentiating into other neural lineage cells such as astrocytes and SCs (Zawadzka et al., 2010; Assinck et al., 2017b; Hackett et al., 2018; Huang et al., 2018). Beyond differentiating into other lineages, OPCs respond to disruptions in tissue homeostasis in a manner reminiscent of microglia. For example, OPCs proliferate and extend their processes to surround local injury sites (Hughes et al., 2013), albeit the kinetics of this process extension are slower than microglia (Davalos et al., 2005; Nimmerjahn et al., 2005). OPCs also migrate to areas of injury and proliferate to compensate for the loss of adjacent OPCs, again reminiscent of microglia. The high proliferative capacity and robust response to tissue injury suggest OPCs themselves may be major regulators of repair. Indeed a number of studies now indicate that the response of OPCs is critical for modulating inflammation, altering glial scarring and potentially regulating angiogenesis. The subsequent sections will review the evidence that OPCs are subverted to directly perform additional functions in CNS repair with an emphasis on how modulating these functions may offer new therapeutic strategies.

### Inflammatory Nature of Oligodendrocyte Progenitor Cells

The rapid proliferation and migration to areas of tissue damage leave OPCs ideally situated to regulate subsequent immune responses. OPCs express many microglia-enriched genes but often at lower levels. For example, the homeostatic microglia marker CX3CR1 is expressed by OPCs (Voronova et al., 2017). Accordingly, OPCs express a number of critical modulators of the inflammatory response including IL-33 and the low-affinity Fc receptor (Fcgr2b) during EAE (Falcão et al., 2018), the latter of which is normally only expressed by microglia (Zhang et al., 2014). Transcriptomic analyses of OPCs reveal an increase in expression of the inflammasome-associated cytokines IL-1β and the chemokine CCL2 during demyelination (Moyon et al., 2015). It should be noted, however, that OPCs were isolated by Moyon et al. (2015) based on PDGFRα expression, which is also enriched in a population of pericytes (Assinck et al., 2017b), so these results should be interpreted with caution. However, recent single-cell RNA sequencing of OPCs during EAE (Falcão et al., 2018) and in MS (Jakubzick et al., 2017) confirms that these cells express a number of proinflammatory genes typically restricted to microglia/macrophages (Butovsky et al., 2014; Zhang et al., 2014). For example in response to IFNγ, OPCs go on to express the antigen presentation molecules MHC-I/II (Falcão et al., 2018). OPCs use MHCI/II to activate T-cells both *in vivo* and *in vitro* (Falcão et al., 2018; Kirby et al., 2018), and the capacity to cross-present antigens to CD8 T-cells was confirmed *in vivo* (Kirby et al., 2018). Taken together, inflammatory demyelination triggers OPCs to express pro-inflammatory cytokines and subsequently present antigens to T-cells.

Given that OPCs can perpetuate an inflammatory response raises the possibility that they might directly induce damage in autoimmune disease. Indeed, the induction of pro-inflammatory genes in OPCs during autoimmune demyelination may be necessary for subsequent demyelination. Blocking the activation of NF-κB by deleting Act1 in NG2+ glia blocks the development of EAE-induced demyelination and diminishes inflammatory gene expression following the adoptive transfer of MOG_35–55_ Th-17 cells (Kang et al., 2013). Thus, the co-option of OPCs into a pro-inflammatory phenotype during Th-17-mediated autoimmune disease likely drives subsequent tissue damage. Additionally, the co-opting of OPCs to perpetuate an inflammatory response may leave them unable to adequately differentiate into new remyelinating oligodendrocytes, a possibility that remains to be proven. Indeed, the adoptive transfer of autoimmune T-cells (Baxi et al., 2015) or engraftment of lymphocytes from people with MS into mice with LPC-induced lesions (El Behi et al., 2017) slows remyelination, which is consistent with OPC immune activities impairing their differentiation. Likewise, following cuprizone demyelination, the transfer of effector T cells or IFNγ impairs oligodendrocyte differentiation and is associated with the increased presentation of antigens by OPCs to CD8+ T cells (Kirby et al., 2018).

This co-option of OPCs into an inflammatory phenotype during autoimmune demyelination raises the intriguing possibility that the pro-inflammatory OPC phenotype likely drives tissue damage and may impair effective remyelination. Current FDA-approved therapeutics for MS target autoimmune damage in the CNS, and as such, they may have an unexpected benefit to diminish the co-option of OPCs, therefore, “freeing” them so that they can effectively remyelinate. In accordance, both the switch of microglia to a less inflammatory phenotype (Miron et al., 2013) and the transfer of regulatory T-cells promote remyelination following chemical demyelination (Dombrowski et al., 2017).

### Oligodendrocyte Lineage Cells During Glial Scarring

When the extent of trauma or tissue injury necessitates glial scar formation, OPCs’ role is to modify other cells that contribute to scar formation such as astrocytes or pericytes (Hackett et al., 2016; Huang et al., 2018) and in some cases directly such as astrocytes (Rodriguez et al., 2014; Hesp et al., 2018). With a milder injury, OPCs have a much-reduced capacity to differentiate into astrocytes (Zawadzka et al., 2010). If proliferating NG2+ cells—which are composed of OPCs, pericytes, SCs and activated microglia/macrophage following traumatic SCI (McTigue et al., 2006)—are depleted, glial scar formation is dramatically impaired (Hesp et al., 2018). The result of NG2+ cell depletion is prolonged hemorrhage, larger lesions, and more pronounced edema (Hesp et al., 2018). Taken together, the proliferation and activation of NG2+ glia, which prominently includes OPCs, is necessary for proper formation of the glial scar that restricts ongoing secondary damage to tissue following traumatic injury. Given that scar formation is associated with ECM molecules inhibitory to remyelination, this raises the intriguing possibility that OPCs indirectly regulate ECM molecules known to inhibit remyelination.

### Oligodendrocyte Lineage Cells Interact With and Regulate the Vasculature

During development, OPCs migrate along blood vessels in order to successfully distribute throughout the parenchyma, a step that is necessary for their subsequent differentiation into myelinating oligodendrocytes (Tsai et al., 2016). The secretion of stromal cell-derived factor 1 (SDF1) by endothelial cells attracts OPCs by binding to CXCR4, which is abundantly expressed on OPCs when Wnt-tone is high (Tsai et al., 2016). However, signaling between OPCs and endothelial cells is bidirectional, and given OPCs’ location along blood vessels in development (Tsai et al., 2016), they are ideally positioned to sense oxygen levels and regulate angiogenesis. OPCs sense oxygen tension within the parenchyma *via* HIF1/2α (Yuen et al., 2014), a protein sensor that is stabilized under hypoxic conditions (Wang et al., 1995; Majmundar et al., 2010). Experimentally stabilizing HIF in OPCs—and therefore mimicking hypoxia for these cells—promotes angiogenesis in both the cortex and corpus callosum likely through the secretion of soluble factors (Yuen et al., 2014). A mechanism emerges in which endothelial cells are necessary for the initial migration of OPCs throughout the parenchyma, which in turn, drive angiogenesis until oxygen tension is sufficient to support proper oligodendrocyte differentiation and myelination (Yuen et al., 2014; Tsai et al., 2016). Paracrine signaling between OPCs and endothelial cells is therefore critical for proper developmental myelination.

Interestingly, recent studies suggest the angiogenic activities of OPCs can be subverted under pathological conditions. In development, heightened OPC clustering along blood vessels disrupts BBB function resulting in lymphocyte trafficking in the CNS parenchyma. Like development (Tsai et al., 2016), OPCs migrate along blood vessels during remyelination (Niu et al., 2019). In active MS lesions, OPCs become trapped and cluster along blood vessels. It is unclear if this heightened clustering is due to local hypoxia within MS lesions or inflammation. Cytokines like IL-1β stimulates OPCs to secrete factors that promote angiogenesis *in vitro*, suggesting inflammation may also regulate OPC-dependent angiogenesis. Excessive Wnt signaling in OPCs not only induced clustering of OPCs but also increased the expression of Wif1, which suppresses tight junction formation in endothelial cells (Niu et al., 2019). Indeed hypoperfusion are present in both the NAWM and NAGM in MS potentially inducing a hypoxic environment (Law et al., 2004; Varga et al., 2009). Inflammation may also subvert OPCs to associate with blood vessels and block their ability to fully differentiate in MS. Given that excessive interactions between OPCs and blood vessels during development are sufficient to trigger pathological immunity, it will be interesting to examine if this interaction promotes pathology in models of inflammatory demyelination like EAE.

**Figure 3:**
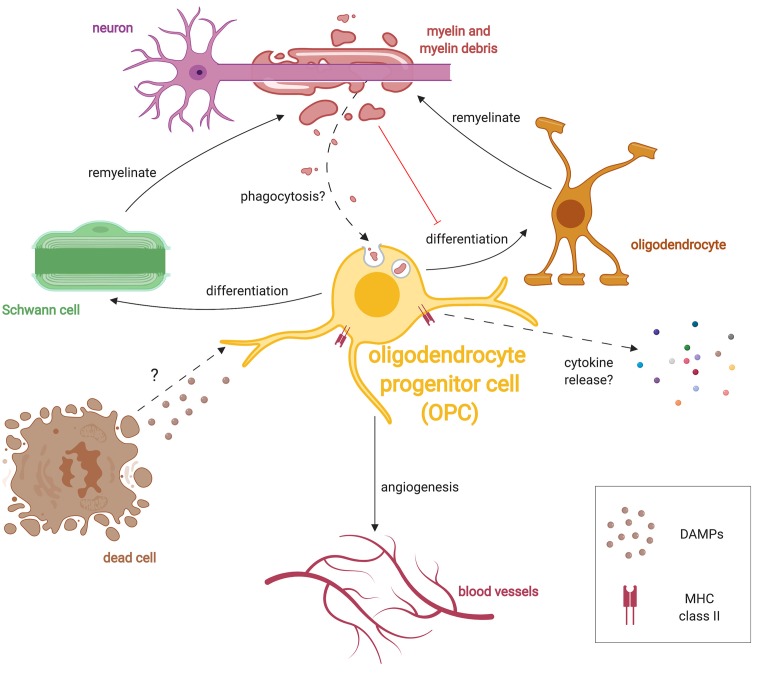
Multiple roles of OPCs during remyelination. After damage, OPCs respond to injury by extending processes, migrating and proliferating following injury presumably in response to unknown DAMPs. OPCs can differentiate into either oligodendrocytes or SCs following demyelination. OPCs also respond to hypoxic conditions and promote angiogenesis. Similar to microglia, OPCs have been shown to phagocytose debris in culture, and may also do so *in vivo*. Given that myelin debris inhibits OPC lineage progression, phagocytosis of debris may subvert OPCs until debris clearance is completed. OPCs in the diseased state are also known to express the antigen-presenting protein, MHC-II, and presumably release specific cytokines which alter the immune response.

Taken together, OPCs are increasingly being recognized for their roles other than differentiating into oligodendrocytes during remyelination (Figure 3). OPCs morphologically respond to injury in a way comparable to microglia, they secrete cytokines, and they regulate scar formation. In these capacities OPCs are an alternative CNS-innate immune cell. OPCs are also responsive to oxygen levels and are important regulators of angiogenesis.

## Remyelination Declines With Age

Remyelination, as with any regenerative process, declines in efficiency with age (Shields et al., 1999; Sim et al., 2002; Ruckh et al., 2012). OPC proliferation and differentiation, which are necessary processes for remyelination, are reduced with age (Sim et al., 2002). In fact, dorsally-derived OPCs in the spinal cord are the predominant source of remyelination in young mice, but contribute less in aged animals (Crawford et al., 2016b). Age can intrinsically alter OPCs directly as old OPCs display a different global DNA methylation profile that alters and/or diminishes their response to pro-remyelinating factors (Zhou et al., 2019). Aging also decreases the recruitment of histone deacetylases (HDACs) in OPCs—epigenetic regulators of gene expression—that leads to a decrease in remyelination efficiency. In OPCs, HDACs downregulate the expression of many transcription factors that inhibit OPC differentiation, including Hes5 and Sox2. They, therefore, regulate remyelination by controlling OPC differentiation (Liu et al., 2006; Shen et al., 2008).

Age-impaired remyelination is also due to impaired innate immune cell function. The removal of inhibitory myelin debris by microglia/macrophages is impaired by aging (Natrajan et al., 2015; Safaiyan et al., 2016; Cantuti-Castelvetri et al., 2018; Rawji et al., 2018). In LPC-induced lesions, aging microglia/macrophages display a decrease in microenvironment surveillance activity and a decrease in phagocytic activity (Rawji et al., 2018). Furthermore, aging microglia/macrophage lysosomal degradation of myelin is diminished as reflected by the accumulation of myelin proteins in microglial lysosomes of aging mice (Safaiyan et al., 2016). This accumulation of myelin debris inside of microglia could be due to a deficiency in reverse cholesterol transport as remyelination efficiency increased when mice with LPC-induced focal demyelination were treated with HβCD, a drug that stimulates cholesterol efflux from cells (Cantuti-Castelvetri et al., 2018). These findings raise the possibility that targeting the phagocytic pathway of microglia/macrophages could improve the efficiency of the myelin debris clearance.

Why myelin debris clearance declines are likely multifaceted? Recently, a CRISPR-Cas9 knockout screen identified CD22 as a negative regulator of microglia phagocytosis that increases with age (Pluvinage et al., 2019). Many factors, such as Retinoid-X-Receptor-α (RXR-α), fractalkine receptor CX3CR1 and Galectin-3 (Gal-3) can also influence the ability of microglia to phagocytose (Lampron et al., 2015; Natrajan et al., 2015; Reichert and Rotshenker, 2019). RXR-α is a ligand-activated transcription factor that controls a variety of genetic programs, including immune cell-related functions (Dawson and Xia, 2012). Natrajan et al. (2015) demonstrate that RXR-α knockout from young macrophages in mice LPC-induced lesion delays myelin debris clearance and remyelination, and also that stimulating RXR-α in monocytes from MS patients *in vitro* improves this normally inefficient myelin debris phagocytosis. There is now an ongoing clinical trial using an RXR-α agonist (EudraCT number: 2014-003145-99). Similarly, the chemokine receptor CX3CR1—enriched in microglia—is critical for myelin debris clearance following cuprizone-induced demyelination (Lampron et al., 2015). Ruckh et al. (2012) found that in the LPC-induced demyelination model, the systemic circulatory system of young mice can restore the efficiency of OPC proliferation, differentiation, and remyelination in aged mice to levels near those observed in young mice. They were able to show these differences by joining the circulatory systems of young and old mice in pairs through heterochronic parabiosis. This rejuvenation of remyelination suggests that reversing the peripheral immune system can partially restore age-dependent decline in remyelination potential. In fact, several human trials such as the Stanford Parkinson’s Disease Plasma Study (NCT02968433) infuse plasma from young people to diseased patients. However, a proper evaluation of the blood-borne rejuvenating factors is needed before any similar trial can be conducted for demyelinating diseases such as MS.

Peripheral changes during aging can also impart effects onto innate immune cells such as microglia. For example, peripheral blood from aged mice is sufficient to lower the levels of type II interferon in the choroid plexus, which is linked to cognitive decline, a common symptom observed in MS patients (Franklin et al., 1989; Baruch et al., 2014). Microglia also have interferon phenotypic changes during aging, in this case, driven in part by higher levels of IFN-I cytokines (Deczkowska et al., 2017) that likely come from peripheral sources. Furthermore, retro-orbital injection of plasma from aging mice to young mice is sufficient to activate microglia (Yousef et al., 2019). Peripheral immune changes are therefore important in regulating the innate immune system in the CNS.

## Conclusion

Remyelination involves a complex interplay between the oligodendrocyte lineage cells that produce the myelin, the astrocytes and the other innate immune cells that regulate the microenvironment of remyelination. Microglia and CNS-infiltrating macrophages regulate the lesion environment and make it suitable for remyelination by activities such as the removal of inhibitory myelin debris. Astrocytes secrete growth factors and provide key lipids species, but also produce inhibitory ECM molecules and the Notch ligand Jagged. OPCs are the key source of remyelinating oligodendrocytes but are now recognized to also regulate angiogenesis, respond to injury and secrete cytokines suggesting they may also be a brain resident immune cell. Given that boosting remyelination spares venerable axons (Mei et al., 2016), more work is required to examine how these cells interact with one another during remyelination.

## Author Contributions

CB, KR, GD and JP drafted and reviewed this manuscript. MH and CB constructed the figures. JP supervised the drafting of this manuscript.

## Conflict of Interest

The authors declare that the research was conducted in the absence of any commercial or financial relationships that could be construed as a potential conflict of interest.
